# Sulfur Hexafluoride 20% versus Lactated Ringer’s Solution for Prevention of Early Postoperative Vitreous Hemorrhage after Diabetic Vitrectomy

**Published:** 2010-04

**Authors:** Fereydoun Farrahi, Mostafa Feghhi, Biuk Bagherzadeh, Mahmood Latifi

**Affiliations:** Department of Ophthalmology, Jundishapur University of Medical Sciences, Ahvaz, Iran

**Keywords:** Diabetic Vitrectomy, SF6, Early Postoperative Vitreous Hemorrhage, Lactated Ringer’s Solution

## Abstract

**Purpose:**

To compare the hemostatic effect of sulfur hexafluoride 20% (SF6 20%) with lactated Ringer’s solution for prevention of early postoperative vitreous hemorrhage following diabetic vitrectomy.

**Methods:**

In a prospective randomized clinical trial, 50 eyes undergoing diabetic vitrectomy were divided into two groups. At the conclusion of surgery, in one group the vitreous cavity was filled with SF6 20% while in the other group lactated Ringer’s solution was retained in the vitreous cavity. The two groups were compared for the rate of early postoperative vitreous hemorrhage.

**Results:**

The incidence of vitreous hemorrhage was lower in the SF6 group than the Ringer’s group 4 days (20% vs 68%, P=0.001), 7 days (24% vs 60%, P=0.01) and 4 weeks (16% vs 40%, P=0.059) after vitrectomy.

**Conclusion:**

In comparison with lactated Ringer’s solution, SF6 20% had a significant hemostatic effect especially in the early postoperative period after diabetic vitrectomy and reduced the incidence of vitreous hemorrhage.

## INTRODUCTION

Vitreous hemorrhage is one of the most serious complications of vitrectomy for proliferative diabetic retinopathy. The incidence of this complication ranges from 8% to 75%.^1^–^6^ It occurs most often in the early postoperative period and may be due to residual hemorrhage or bleeding from cut edges of fibrovascular tissue dissected during the operation. Advances in surgical technique and use of more sophisticated instrumentation including endodiathermy and endolaser have reduced the incidence of this condition to about 25%.^7^

Postvitrectomy vitreous hemorrhage decreases visual acuity and impairs fundus visualization, it interferes with laser photocoagulation and detection of other retinal complications, furthermore it can predispose to cellular proliferation and may also increase intraocular pressure (IOP).^8^ Various substances including epsilon-aminocaproic acid,^9^ sodium hyaluronate,^10^,^11^ air,^12^ thrombin,^13^ tranexamic acid,^14^,^15^ sulfur hexafluoride (SF6),^16^ perfluoropropane (C3F8),^17^ and bevacizumab^18^,^19^ have been used to prevent vitreous hemorrhage after diabetic vitrectomy.

Thompson et al^20^ used SF6 for tamponade of suspected retinal breaks or to prevent early postoperative vitreous hemorrhage. However, they did not report any data on the effect of the gas on the incidence of postoperative vitreous hemorrhage. Koutsandrea et al^16^ compared the hemostatic effect of SF6 20% versus balanced salt solution (BSS) in diabetic vitrectomy.

Regarding the lack of data on the incidence of vitreous hemorrhage following diabetic vitrectomy using lactated Ringer’s solution, this pilot study was performed to evaluate the hemostatic effect of SF6 20% as compared to lactated Ringer’s solution in the early postoperative period following diabetic vitrectomy.

## METHODS

In a prospective randomized clinical trial, 67 eyes of 67 patients undergoing diabetic vitrectomy who met the inclusion criteria were randomly divided into two groups using a random number table. Informed consent was obtained from all patients preoperatively. Indications for vitrectomy were nonclearing vitreous hemorrhage and progressive fibrovascular proliferation unresponsive to panretinal laser photocoagulation. Patients with orthopedic or systemic problems who were unable to maintain prone position, monocular patients, subjects who were obliged to travel by air immediately after the operation, those who required concomitant cataract surgery, as well as patients with rubeosis iridis, extensive tractional retinal detachment with or without rhegmatogenous retinal detachment were excluded. Patients who required silicone oil injection intraoperatively were also excluded from the study. Seventeen eyes were excluded due to intraoperative use of silicone oil. Eventually 50 eyes of 50 patients fulfilled the inclusion and exclusion criteria of the study and were analyzed.

All eyes underwent standard three port pars plana deep vitrectomy by two surgeons; complete posterior vitreous detachment was induced and delamination and segmentation of preretinal fibrovascular tissue was performed. We decided to use lactated Ringer’s solution instead of BSS during diabetic vitrectomy in both groups because BSS contains sodium citrate which has anticoagulant properties and may promote vitreous hemorrhage.^21^ The vitreous cavity was filled with SF6 20% in 25 eyes but lactated Ringer’s solution was retained in the vitreous cavity of 25 other eyes at the end of the procedure. At the conclusion of surgery, the sclerotomies were repaired in the Ringer’s group but fluid was exchanged with SF6 20% in the SF6 group before repairing the sclerotomies. All patients in the SF6 group were instructed to maintain prone position for 7 to 10 days after the operation. Patients were examined 1, 4 and 7 days as well as 4 weeks postoperatively. Follow-up was limited to 4 weeks because SF6 completely clears from the eye and loses its tamponade effect by that time. Postoperative examinations included assessment of visual acuity, slitlamp biomicroscopy, IOP measurement and indirect ophthalmoscopy.

The extent of vitreous hemorrhage was graded using indirect ophthalmoscopy according to the Diabetic Retinopathy Vitrectomy Study (DRVS) as follows:^22^ 0, no vitreous hemorrhage; 1, mild vitreous hemorrhage with visible fundus details; 2, moderate vitreous hemorrhage with no visible fundus details but with an orange fundus reflex; and 3, severe vitreous hemorrhage with no fundus details and no fundus reflex. Data analysis was performed using Chi-square test for frequency values and *t*-test for mean values; P values <0.05 were considered significant.

## RESULTS

Fifty eyes of 50 patients were enrolled and randomized to SF6 20% (25 eyes) versus lactated Ringer’s solution (25 eyes). Mean age was 53.8±11.3 (range, 25–77) years in the SF6 group and 51.4±12.5 (range, 26–74) years in the Ringer’s group (P=0.417). Seventeen (68%) patients in the Ringer’s group and 13 (52%) patients in the SF6 group were female (P=0.248). The incidence of vitreous hemorrhage was higher in the Ringer’s group at all postoperative visits (Table 1).

Eleven (22%) patients had systemic hypertension preoperatively and were receiving antihypertensive agents. The incidence of vitreous hemorrhage during the follow-up period was 54.5% (6 cases) among hypertensive patients and 43.5% (16 cases) among normotensive patients (P=0.425). Forty-two eyes were phakic of which 3 eyes (6%), including two eyes in the SF6 group and one eye in the Ringer’s group, developed cataracts postoperatively. Mean IOP was 12.13±1.24 mmHg in the SF6 group and 12.16±0.34 mmHg in the Ringer’s group (P=0.951) 4 weeks postoperatively. Forty-one (82%) patients had type II and 9 (18%) patients had type I diabetes mellitus. The incidence of vitreous hemorrhage during the follow-up period was 46.3% (19 cases) in type II diabetic patients and 44.4% (4 cases) in type I patients (P=0.87).

Visual acuity was hand motions or worse in 26 (52%) patients and better than hand motions but less than 6/120 in 24 (48%) patients preoperatively with no significant difference between the two groups (data not presented). At final visit, 11 (22%) eyes had visual acuity worse than 6/120, including 7 (28%) eyes in the Ringer’s group and 4 (16%) eyes in the SF6 group; 13 (26%) eyes had visual acuity between 6/120 and 6/60, including 8 (32%) eyes in the Ringer’s group and 5 (20%) eyes in the SF6 group; and 26 (52%) eyes had visual acuity better than 6/60, including 10 (40%) eyes in the Ringer’s group and 16 (64%) eyes in the SF6 group. The difference in postoperative visual acuity between the study groups was not statistically significant (P=0.235).

## DISCUSSION

Despite laser treatment for proliferative diabetic retinopathy, the ocular sequelae of diabetes mellitus remain one of the most common indications for deep vitrectomy. Postoperative vitreous hemorrhage is a common complication of diabetic vitrectomy with a reported incidence of up to 75%. Advances in surgical technique and instrumentation including endodiathermy and endolaser photocoagulation have reduced the incidence of this complication to 25%.^7^ Postoperative vitreous hemorrhage is associated with the following: (1) reduced vision which is annoying especially in monocular patients, (2) interference with fundus examination which may preclude diagnosis of postoperative problems such as retinal detachment, (3) interference with laser therapy, (4) ghost cell glaucoma, (5) introduction of platelet-derived growth factor (PDGF) and fibrinogen into the vitreous cavity causing fibrovascular proliferation, and (6) need for repeat surgery.

Various substances have been used to prevent vitreous hemorrhage after diabetic vitrectomy. De Bustros et al^9^ used epsilon-aminocaproic acid and found that it significantly reduced postoperative vitreous hemorrhage during the period of hospitalization, but its effect was not statistically significant later than 2 weeks following the operation. Falk et al^10^ and Packer et al^11^ used sodium hyaluronate in the vitreous cavity at the end of phakic diabetic vitrectomy which appeared to control vitreous hemorrhage in the immediate postoperative period but entails potential side effects such as elevated IOP. In a randomized study, Joondeph et al^12^ evaluated the hemostatic effect of air versus fluid in diabetic vitrectomy in an animal model and reported no statistically significant difference between them, which is in contrast to our study. This may be due to the short half-life and weak tamponade provided by air in the vitreous cavity. Laatikainen et al^14^ and Ramezani et al^15^ evaluated the effect of tranexamic acid in preventing vitreous hemorrhage after diabetic vitrectomy and reported no statistically significant effect.

Koutsandrea et al^16^ investigated the hemostatic effect of SF6 versus BSS in diabetic vitrectomy and reported no significant difference. The authors’ definition of vitreous hemorrhage was poor visualization of retinal vessels and fundus details and a red-orange or dark red fundus reflex, thereby they seem to have ignored mild vitreous hemorrhage.

Yang et al 17 evaluated the hemostatic effect of intravitreal injection of C3F8 10%, a long acting gas, on the occurrence of early postoperative vitreous hemorrhage in patients undergoing diabetic vitrectomy and concluded that it may be a useful adjunct to vitrectomy for proliferative diabetic retinopathy and may reduce the rate of recurrent vitreous hemorrhage in the early postoperative period (P=0.02). Ahmadieh et al^18^ and Modarres et al^19^ in separate studies evaluated intravitreal injection of bevacizumab before diabetic vitrectomy and both investigators concluded that this modality may decrease the incidence of early post-vitrectomy hemorrhage.

In our study, we employed lactated Ringer’s solution instead of BSS, because the latter contains citrate which can promote bleeding during and after surgery. Contrary to Koutsandrea et al,^16^ we recorded all types of vitreous hemorrhage according to the DRVS study.^22^ The hemostatic effect of SF6 was significant during the first postoperative week, however this effect declined reaching borderline significance four weeks thereafter. However, we believe that this effect was clinically significant and had the sample size been larger, the observed difference would also have reached statistical significance at this time point. Further studies with larger sample size are needed to draw a definite conclusion. Diminution of the difference between the incidence of vitreous hemorrhage between the study groups at 28 days is probably due to elimination of SF6 from the eye after four weeks. The current report was a pilot study and despite our attempts to increase the number of cases, the absence of a predetermined sample size and the limited number of cases is a shortcoming. The surgeons were not masked to the intervention throughout the operation and this may be a source of bias.

It summary we may conclude that SF6 20% in the vitreous cavity provides temporary tamponade and reduces the incidence of postoperative diabetic vitreous hemorrhage; disadvantages include the potential for inducing cataracts and prone positioning.

## Figures and Tables

**Table 1:** Incidence and severity of vitreous hemorrhage in the study groups during the follow-up period

Groups		Vitreous hemorrhage			

		Mild	Moderate	Severe	Total
**4 days:**	SF6	2	0	3	5 (20%)
	Ringer’s	4	7	6	11 (68%)
P value		-	-	-	0.001

**7 days:**	SF6	3	0	3	6 (24%)
	Ringer’s	9	3	3	15 (60%)
P value		-	-	-	0.01

**4 weeks:**	SF6	1	1	2	4 (16%)
	Ringer’s	3	2	5	10 (40%)
P value		-	-	-	0.059
